# Experiences of stigma and discrimination of people with schizophrenia in India

**DOI:** 10.1016/j.socscimed.2014.10.035

**Published:** 2014-12

**Authors:** Mirja Koschorke, R. Padmavati, Shuba Kumar, Alex Cohen, Helen A. Weiss, Sudipto Chatterjee, Jesina Pereira, Smita Naik, Sujit John, Hamid Dabholkar, Madhumitha Balaji, Animish Chavan, Mathew Varghese, R. Thara, Graham Thornicroft, Vikram Patel

**Affiliations:** a Centre for Global Mental Health, Institute of Psychiatry, Psychology & Neuroscience, King's College, London, UK; b Schizophrenia Research Foundation (SCARF), Chennai, India; c Samarth, Chennai, India; d Centre for Global Mental Health, London School of Hygiene and Tropical Medicine, London, UK; e MRC Tropical Epidemiology Group, London School of Hygiene and Tropical Medicine, London, UK; f Sangath, Goa, India; g Parivartan, Satara, India; h Nirmittee, Satara, India; i NIMHANS, Bengaluru, India

**Keywords:** India, Stigma, Discrimination, Mental illness, Schizophrenia, Mixed methods

## Abstract

Stigma contributes greatly to the burden of schizophrenia and is a major obstacle to recovery, yet, little is known about the subjective experiences of those directly affected in low and middle income countries. This paper aims to describe the experiences of stigma and discrimination of people living with schizophrenia (PLS) in three sites in India and to identify factors influencing negative discrimination.

The study used mixed methods and was nested in a randomised controlled trial of community care for schizophrenia. Between November 2009 and October 2010, data on four aspects of stigma experienced by PLS and several clinical variables were collected from 282 PLS and 282 caregivers and analysed using multivariate regression. In addition, in-depth-interviews with PLS and caregivers (36 each) were carried out and analysed using thematic analysis.

Quantitative findings indicate that experiences of negative discrimination were reported less commonly (42%) than more internalised forms of stigma experience such as a sense of alienation (79%) and significantly less often than in studies carried out elsewhere. Experiences of negative discrimination were independently predicted by higher levels of positive symptoms of schizophrenia, lower levels of negative symptoms of schizophrenia, higher caregiver knowledge about symptomatology, lower PLS age and not having a source of drinking water in the home. Qualitative findings illustrate the major impact of stigma on ‘what matters most’ in the lives of PLS and highlight three key domains influencing the themes of 'negative reactions' and ‘negative views and feelings about the self’, i.e., ‘others finding out’, ‘behaviours and manifestations of the illness’ and ‘reduced ability to meet role expectations’.

Findings have implications for conceptualising and measuring stigma and add to the rationale for enhancing psycho-social interventions to support those facing discrimination. Findings also highlight the importance of addressing public stigma and achieving higher level social and political structural change.

## Highlights

• Internalised experiences of stigma were reported more commonly than discrimination.
• A range of negative reactions beyond ‘discrimination’ led to feelings of devaluation.
• Negative discrimination was predicted by illness features, e.g., positive symptoms.
• Negative reactions were also linked to reduced ability to meet role expectations.
• What mattered most to PLS was to achieve role expectations in marriage and work.

## 1 Introduction

The stigma associated with mental illness contributes significantly to the burden of schizophrenia. Subjective accounts of persons affected by mental illness testify that its effects are often perceived as more burdensome and distressing than the primary condition itself (Thornicroft, 2006). The term stigma refers to “a social devaluation of a person” (Thara and Srinivasan, 2000, p. 135) due to an “attribute that is deeply discrediting” (Goffmann, 1963, p.3), and can be conceptualised as consisting of “problems of ignorance, prejudice, and discrimination”(Thornicroft, 2006, p. 182). Discrimination leads to disadvantages in many aspects of life including personal relationships, education and work. As a result of internalised stigma, some people with mental illness may further accept the discrediting prejudices held against them and lose self-esteem, leading to feelings of shame, a sense of alienation and social withdrawal (Livingston and Boyd, 2010, Ritsher et al., 2003). Therefore, people with mental illness may expect to be treated in a discriminatory way (‘anticipated discrimination’) and try to hide their illness or stop themselves from taking up opportunities (Ritsher et al., 2003, Thornicroft, 2006).

While it is widely accepted that stigma constitutes a universal phenomenon, experiences of stigma and discrimination are local (Murthy, 2002). Yang et al. point out that “across cultures, the meanings, practices and outcomes of stigma differ, even when we find stigmatisation to be a powerful and often preferred response to illness, disability and difference” (Yang et al., 2007, p. 1528).

Although there is now a large evidence base on descriptive aspects of stigma, the great majority of these studies have been carried out in high-income settings (Livingston and Boyd, 2010, Mestdagh and Hansen, 2014). Given the importance of context-specific factors in shaping stigma, research is needed to understand which aspects of the experience of stigma are most common and burdensome in the Indian context and which determinants are relevant and potentially amenable. The aim of our study was to contribute to such context-specific understanding of stigma and to inform the design of future anti-stigma interventions in India.

Similar to studies in other parts of the world (Thornicroft, 2006), research from India has illustrated high levels of stigmatising attitudes towards PLS among community members and health staff (see (Loganathan and Murthy, 2008) for a summary). Impact of stigma on help-seeking and other aspects of health has been shown to be high (Shidhaye and Kermode, 2013). However, much less is known about the subjective experiences of PLS in India. In one particularly informative Indian study PLS reported being ridiculed, avoided or looked down upon. A few were given stale food, stopped from leaving the house, beaten or hit with stones. Some spoke about lack of respect from family members (Loganathan and Murthy, 2008). Men experienced stigma most strongly in regard to employment, and women in relation to marriage and childbirth (Loganathan and Murthy, 2011). In another study, stigmatising reactions were often enacted by family members and neighbours (Murthy, 2005).

Women's experiences of stigma were also explored in a qualitative study, which involved 76 women with schizophrenia whose marriages had broken. Many had been abandoned by their husbands and only very few received financial support. Some had experienced beating and neglect. Several felt a burden to their parents, which was reinforced by hostility from family members (Thara et al., 2003).

Little is known about the determinants of subjective experiences of stigma associated with in India. While research from high-income countries (HIC) indicates that symptom severity is one of the most consistently identified determinants of experienced stigma (Livingston and Boyd, 2010), research from India on this association is scarce and has shown conflicting results (Charles et al., 2007, Loganathan and Murthy, 2008, Raguram et al., 2004). Yet, the importance of illness features and behaviours in determining social reactions has been highlighted (Raguram et al., 2004, Weiss et al., 2001).

As Yang et al. have noted, a crucial condition for understanding the experience of stigma in different cultural contexts is understanding what is ‘at stake’ or ‘what matters most’ (Yang et al., 2014a, Yang et al., 2014b, Yang et al., 2007). No studies so far have examined stigma in India specifically from this perspective.

This study employed mixed methods to describe the experiences of stigma and discrimination of PLS in three diverse sites in India with a focus on ‘what matters most’ and on information that may be relevant to the development of interventions.

## 2 Methods

### 2.1 Setting

The study was nested in a randomised controlled trial of collaborative community care for people with schizophrenia in India (COPSI Trial) and implemented in three diverse settings in India–in rural Tamil Nadu by the Schizophrenia Research Foundation (SCARF) and in two mixed urban and rural sites in Goa and Maharashtra (Satara) by the NGOs ‘Sangath’, ‘Parivartan’ and ‘Nirmittee’ in collaboration with private psychiatrists (Balaji et al., 2012b, Balaji et al., 2012a, Chatterjee et al., 2011, Chatterjee et al., 2014a, Chatterjee et al., 2014b). In Tamil Nadu, psychiatric care was provided through the rural clinics of SCARF and in Satara and Goa by private psychiatrists. Full characteristics of the study sites have been described elsewhere (Chatterjee et al., 2011).

The study used cross-sectional data collected at entry into the trial (between November 2009 and October 2010) and employed a concurrent mixed methods design, combining quantitative data from all PLS and caregivers in the trial (282 PLS and 282 primary caregivers) and qualitative data from a purposively selected subsample (36 PLS and 36 caregivers). This paper presents integrated study findings on stigma faced by PLS, drawing on quantitative assessments with PLS and qualitative interviews with PLS and caregivers. Findings on caregivers' own experiences of stigma will be reported separately.

### 2.2 Recruitment and sampling

The quantitative study included the total sample of participant dyads recruited for the COPSI trial (*n* = 282), with 105 dyads from Tamil Nadu, 92 from Goa and 85 from Satara. In Goa and Satara, PLS were recruited from the clinical practices of collaborating psychiatrists; in Tamil Nadu, they were identified through a community survey and referred to the clinics of SCARF. For each PLS, one primary caregiver was recruited.

Eligibility criteria were i) 16–60 years old, ii) a primary diagnosis of schizophrenia as per ICD-10 DCR criteria (WHO, 1992) (diagnosed by the treating psychiatrist), iii) illness duration of at least 12 months and an overall moderate severity of the illness based on the Clinical Global Impression-Schizophrenia (CGI-SCH) (Haro et al., 2003) scale and iv) residence within the catchment area for the duration of the study.

A subsample of 36 PLS - caregiver dyads from those already recruited for the trial was selected for participation in the qualitative study component. A purposive sampling technique was applied, aiming to ensure adequate sample variability for PLS gender, severity of illness according to the PANSS (Kay et al., 1987), highest household education level and research site. To facilitate the in-depth study of experienced stigma, there was a slight intentional overrepresentation of PLS reporting higher levels of negative discrimination on the Discrimination and Stigma Scale (DISC) (Brohan et al., 2013).

Informed consent was obtained from all PLS and caregivers taking part, with an additional level of consent provided by those participating in qualitative interviews (Chatterjee et al., in submission; Chatterjee et al., 2011)). Ethics approval was obtained from the Institutional Review Boards at SCARF and Sangath in India, and the Ethics Committees at the London School of Hygiene and Tropical Medicine (approval number 5579) and King's College, London (PNM/08/09-121) in the UK.

### 2.3 Quantitative assessments

Quantitative data were collected on four aspects of PLS’ experience of stigma and discrimination:

‘Negative discrimination’ and ‘anticipated discrimination' were assessed using respective subscales of the Discrimination and Stigma Scale (DISC), a standardised assessment of discrimination which has been employed in HIC and low-and-middle-income countries (LAMIC) including India (Brohan et al., 2013, Thornicroft et al., 2009). Participants were asked whether they had been treated unfairly because of their mental health problems in specified domains of everyday life in the last 12 months, e.g., ‘Have you been treated unfairly by the people in your neighbourhood?’ or whether they had stopped themselves from taking up opportunities because of anticipated discrimination, e.g., ‘Have you stopped yourself from applying for work’? Participants rated their response according to the felt overall severity of their experience (quantitative and qualitative) on a 4-point Likert Scale (‘not at all’, ‘a little’, ‘moderately’, ‘a lot’).

To measure PLS’ experiences of ‘alienation’, we employed the ‘Alienation Subscale’ of the Internalised Stigma of Mental Illness (ISMI) scale (Ritsher et al., 2003). This measure consists of six statements (e.g., ‘I feel out of place in the world because I have a mental illness’) for which agreement is rated on a 4-point Likert scale. A further (single-item) measure was included to assess participants' *‘willingness to disclose the illness’* (TNS UK Care Services Improvement Partnership, 2009).

Symptoms of schizophrenia were measured using the Positive and Negative Syndrome Scale (PANSS) (Kay et al., 1987), administered by two psychiatrists and one psychologist supervised by external experts. To measure caregivers' knowledge about schizophrenia, researchers assessed six domains of understanding (Table 1) using the Knowledge About Schizophrenia Interview (KASI) (Barrowclough and Tarrier, 1992).

**Table 1:** Sample characteristics.

Sample characteristics total sample (*N* = 282 PLS – Caregiver dyads)			
Characteristics of PLS (*N* = 282)		Characteristics of caregivers (*N* = 282)	
**PLS gender**	*n* (%)	**Caregiver gender**	*n* (%)
Male	150 (53.2)	Male	93 (33.0)
Female	132 (46.8)	Female	189 (67.0)
**PLS age (years)**	*n* (%)	**Caregiver age (years)**	*n* (%)
16–24	36 (12.7)	16–34	54 (19.2)
25–34	97 (34.4)	35–44	45 (16.0)
35–44	90 (31.9)	45–54	76 (27.0)
45–54	38 (13.5)	55–64	61 (21.6)
55 or above	21 (7.5)	65 or above	46 (13.3)
**PLS marital status**	*n* (%)	**Type of relationship to PLS**	*n* (%)
Never married	121 (43.4)	Parent	145 (51.4)
Married	121 (43.4)	Spouse	70 (24.8)
Separated/divorced	23 (8.2)	Sibling	36 (12.8)
Widowed	14 (5.0)	Other family member	31 (11.0)
*missing*	*3*		
**PLS occupational status**	*n* (%)	**Caregiver knowledge about schizophrenia**	Mean (SD)
Not income-generating (Unemployed; Housewife)	204 (73.1)	KASI total score (Possible range: 6–24)	13.4 (2.7)
Income-generating	64 (22.9)		
Any other	11 (3.9)	KASI Sub–scores (Possible range: 1–4)	2.1 (0.6)
*missing*	*3*		
Duration of illness (years)	Median (IQ range) 6.3 (6.0–11.0)	Knowledge about diagnosis	2.3 (0.7)
*missing*	*19*		
**Symptom severity (PANSS Scores)**	Mean (SD)	Knowledge about symptomatology	
PANSS total symptom Score (Possible range: 30–210)	75.7 (19.9)	Knowledge about aetiology	1.9 (0.6)
PANSS positive symptom score (Possible range: 7–49)	17.5 (6.7)	Knowledge about medication	2.3 (0.8)
PANSS negative symptom score (Possible range: 7–49)	21.4 (7.5)	Knowledge about course and prognosis	2.0 (0.8)
PANSS general symptom score (Possible range: 16–112)	36.9 (10.1)	Knowledge about management	2.8 (0.9)
Household level characteristics			
**Research site**	*n* (%)	**Highest education level in the household**	*n* (%)
Tamil Nadu	105 (37.2)	up to 8th Standard	32 (11.4)
Satara	85 (30.1)	9th – 12th Standard	114 (40.7)
Goa	92 (32.6)	College or above	134 (47.9)
**Urbanicity**	*n* (%)	**Source of drinking water**	*n* (%)
Rural	195 (69)	Tap water (in the house)	162 (57.5)
Urban	87 (31)	Other source (public tap, river, etc.)	120 (42.6)

Data on a range of socio-demographic variables were obtained, including highest household education level and source of drinking water (as indices of economic deprivation) (International Institute for Population Sciences.). Details of all measures used have been published elsewhere (Chatterjee et al., 2011, Chatterjee et al., 2014a, Chatterjee et al., 2014b).

A standardised process of translation and validation of outcome tools was followed in order to obtain translation that were meaningful in the local context Chatterjee et al., 2011, Chatterjee et al., 2014a, Chatterjee et al., 2014b Measures then underwent additional validation through three focus groups with 8–10 participants each, involving PLS, caregiver and mental health staff representatives, which served to clarify conceptual meanings and derive locally relevant probes. Based on formative study findings, two items of the negative discrimination subscale of DISC version 12, items 4 (relating to housing) and 17 (second of two items relating to mental health staff), were removed as they were rarely endorsed.

Data were collected by a team of three to four trained research assistants in each site using programmed palmtop computers and pen-and-paper methods. Researchers saw participants at their home, or – if preferred by them – at a different location. Care was taken to ensure privacy during these visits.

### 2.4 Qualitative assessments

The qualitative study of experiences of stigma was an open exploration of illness experience, with a particular focus on relationships. Semi-structured in-depth-interviews (DiCicco-Bloom & Crabtree, 2006) were carried out with a purposively selected subsample of 36 PLS and 36 caregivers 2–4 weeks after the quantitative assessments.

The interview guide (Online file A) was developed in a collaborative process involving researchers and clinicians in all sites and was adapted in 27 formative and 24 pilot interviews (Balaji et al., 2012b, Balaji et al., 2012a). Ongoing data analysis served to refine probes and provide feedback to researchers.

Interviews were held by trained researchers, who were local graduates, in local languages (Konkani, Marathi, or Tamil) or English, as preferred by the participants. Care was taken to carry out interviews in private, without family members present. Interviews were audio-recorded and transcribed by the researchers who had conducted them. A verbatim account of the interviews was recorded and supplementary information noted. Transcripts were translated into English by the researchers who had conducted them (Goa) or external translators (Satara and Tamil Nadu). The accuracy of transcription and translation was cross-checked against the audiotape by someone fluent in English and the local language.

### 2.5 Analysis

Statistical analyses were carried out using Stata11(Stata Corp., 2009). The primary outcome, any negative discrimination, was first examined in univariate regression analyses.

To test the hypothesis that symptom severity was significantly associated with experiences of negative discrimination, crude effect measures for PANSS total symptom score and each of its sub-scores were estimated. Potential confounders were identified following an a-priori analytic diagram based upon the existing literature. Variables which were associated with the outcome with a *p*-value of <0.1 in univariate analyses (PLS age, type of relationship to caregiver, source of drinking water, caregiver knowledge about symptomatology and caregiver knowledge about diagnosis), plus the variable PLS gender (a priori) were then tested as to whether they acted as confounders. No confounding was identified; therefore, crude effect estimates were interpreted with regard to the hypothesis.

Next, we aimed to identify the factors independently associated with negative discrimination. The above mentioned variables which were associated with the outcome with a *p*-value of <0.1 were included in the multivariate regression model following a hierarchical approach (Victora et al., 1997). Only factors which remained significant with *p* < 0.10 after adjusting for the other factors in the model were retained in the final model (Table 2; Online file B).

The analysis of qualitative interview data used techniques of thematic analysis (Braun and Clarke, 2006) and began as soon as interviews were available. NVivo 8 (QSR International Pty Ltd., 2008) was used for coding and higher levels of analysis. The analytic process was collaborative and involved the authors and interviewers in all sites. The list of topics covered by the interview guide was derived from a deductive framework based on a literature review, which was set aside once data collection had commenced to allow the process to be as inductive as possible. Thus, analysis did not follow a specific stigma framework but rather aimed to explore the meaning of ‘stigma’ from the perspective of interviewees. First, a set of transcripts were coded independently by researchers using ‘open coding’ (Green and Thorogood, 2004). The group discussed the codes and tentative categories were derived. A further six interviews were coded independently by RP and MK using the revised scheme. Coding was compared, significant differences resolved and definitions clarified.

MK then coded a representative subset of transcripts (*n* = 24 interview pairs) while 12 interview pairs were coded by RP and SK. The scheme was continually developed as analysis progressed to incorporate new codes. A trail of coding steps was maintained.

The process of identifying themes and links in the data started during coding and involved a collaborative review of the material coded and clustering of codes to form categories. Relationships between categories were examined in order to decide which informed the same overarching concepts. Preliminary themes were established and examined for ‘internal homogeneity and external heterogeneity’ (Braun and Clarke, 2006).

The development of the thematic network was inductive and drew upon tentative links among categories and themes which were captured while coding using an additional level of inductive codes that captured statements in which these links were apparent. It was further informed by a log of analytic observations noted during coding, written case summaries and analytic collaborators' meetings.

A preliminary thematic network was developed based on an interim analysis of the first 18 interview pairs coded. This was cross-checked and validated based on the full data set available (*n* = 36). The final thematic network (Fig. 2) illustrates the results of this substantially data-driven process.

**Fig. 1:**
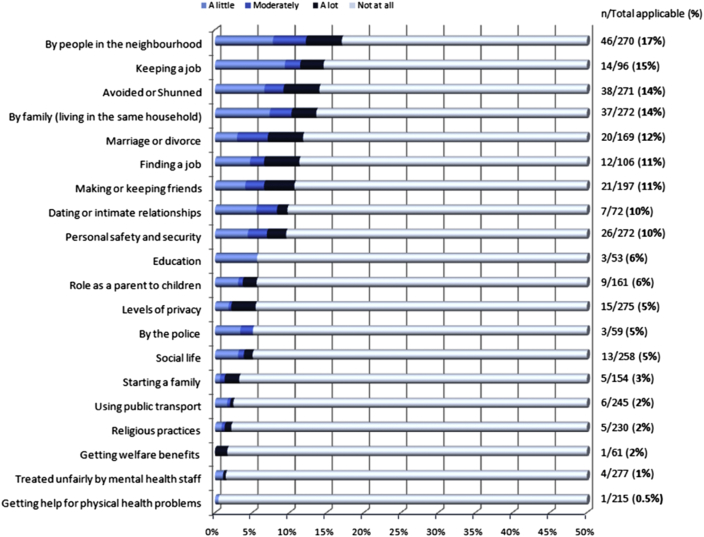
Negative discrimination item percentages.

**Fig. 2:**
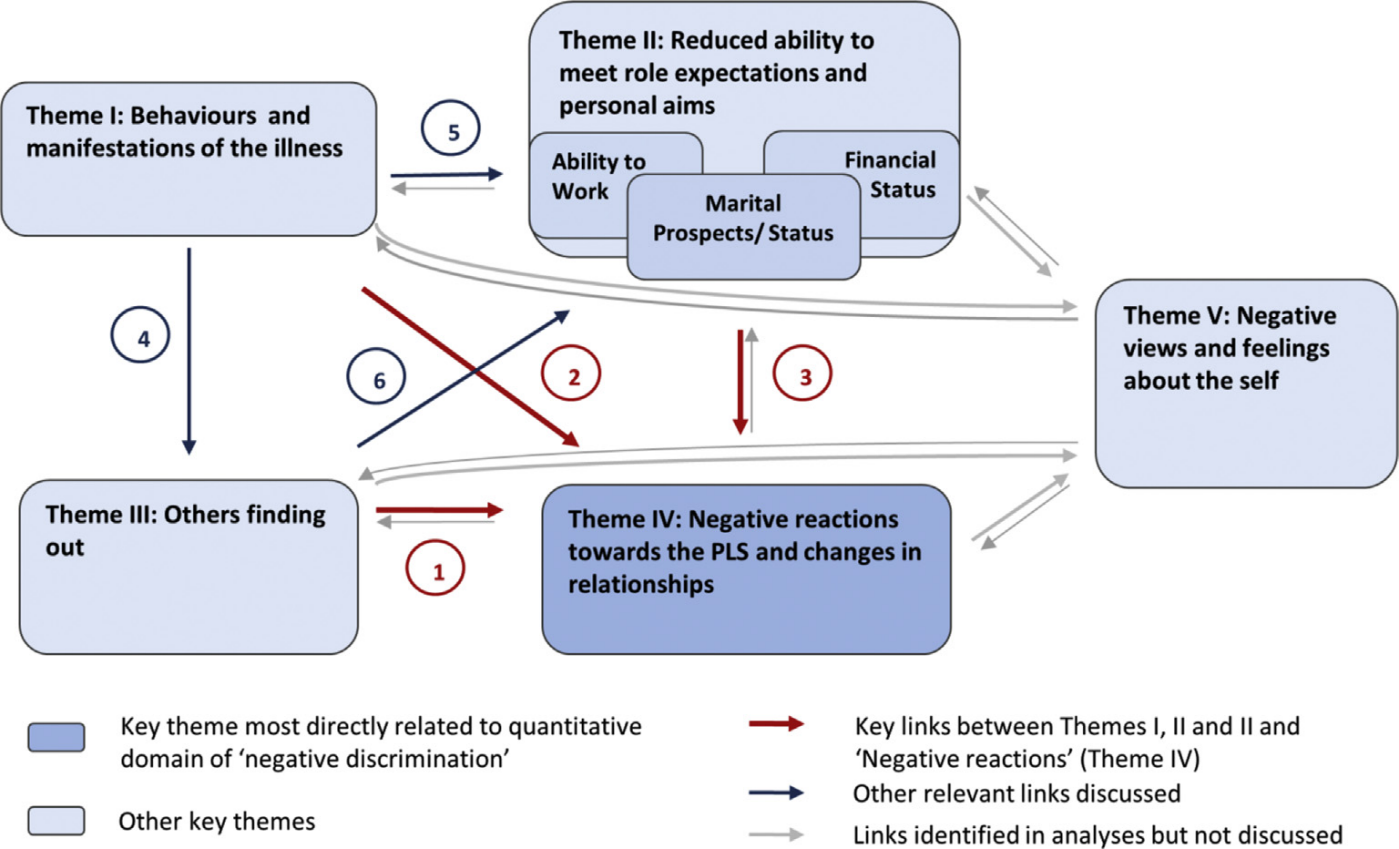
Thematic network – Negative reactions towards the PLS and links to other domains.

## 3 Results

### 3.1 Sample description

The sample for quantitative analyses consisted of 282 dyads comprising one PLS and his/her primary caregiver (Table 1).

The qualitative sample (*n* = 36 dyads) consisted of a subgroup of the participants already recruited for the trial. It comprised 18 male and 18 female PLS (and their caregivers), with 14 dyads from Tamil Nadu, 12 from Satara and 10 from Goa. The qualitative sample was similar to the quantitative (total) sample with regards to key socio-demographic and clinical characteristics. PLS who reported higher experiences of negative discrimination on the DISC were purposively overrepresented: 29% of those selected compared to 10% of the total sample reported experiences of negative discrimination in four or more life areas covered by the DISC.

### 3.2 Findings on experiences of stigma and discrimination

This section presents the findings on four quantitative measures of PLS’ experiences of stigma alongside qualitative findings on related qualitative subthemes. Thus, we do not attempt to summarise the entire set of qualitative findings, but instead describe those themes which most directly correspond to the concepts measured quantitatively in order to triangulate and explore these findings further. To achieve a comprehensive understanding of PLS’ experiences of stigma without premature categorisations, we decided to include all forms of ‘uncomfortable reactions’ reported, irrespective of their attribution.

The four domains explored in this mixed-methods approach were determined a priori by the four aspects of stigma measured quantitatively *(the names of corresponding qualitative themes are in italics):*

#### 3.2.1 Negative discrimination/*Experiences of negative reactions*

Overall, 42% of PLS reported that, in the last year, they had experienced negative discrimination in one or more of the 20 life areas covered by the DISC in the last year. Close to 20% reported discrimination in one area of life, 11% in two to three areas and 10% in more than four areas.

The proportion of PLS who reported any negative discrimination in a given area of life ranged from 0.5 to 17.0%. Discrimination was reported more commonly from sources closer to the PLS (e.g., neighbours, family members or friends) or relating to work, than from other institutional sources (e.g., from the police, or from healthcare staff) (Fig. 1).

In qualitative interviews, negative social reactions, of varying levels of severity, were reported in nearly all interview transcripts. The most important types of negative reactions (in terms of salience and frequency) were being ‘*avoided by others’,* being ‘*treated differently or with lack of respect’, ‘teasing or negative comments’* or *‘angry reactions’,* e.g., being *‘scolded or shouted at’.* A small number of PLS reported *physical aggression,* e.g.*, beating,* or incidents of *sexual abuse* or *neglect.* Of note, PLS were often concerned about *perceived reactions and perceived status loss*, even in the absence of experiences of enacted discrimination, e.g., they would express distress at their perception that others were *‘gossiping’* or *‘looking down upon them’*.

A woman from Tamil Nadu described her experience as follows:

*“Earlier [my relatives] would visit me. We would meet up and speak. They would regularly visit me. Now that I have this illness, no one comes regularly to meet me ( … ) even if they come, they will speak to them [other family members]; they will not speak to me.”*

Most interviewees reported both positive, supportive reactions and negative reactions from family members with whom they lived. The negative reactions experienced from within the family were *‘scolding, shouting and derogatory comments’, ‘distancing and loss of affection’*, *‘loss of status and respect in the family’, ‘restrictions’* (being stopped from going out; in one case chaining during acute phases of the illness) and, in several families, *‘beating’.* Several PLS reported feeling distressed and devalued as a consequence.

To better understand how PLS appraised these negative reactions, we asked about how they thought others should be treating them. Interviewees were often distressed at the negative reactions they experienced, and some stated clearly that they thought it was not right that they were *‘treated differently’*. A minority used terms akin to ‘stigma’ and ‘discrimination’ to describe their experiences, or agreed that those terms applied to their experiences when asked about this at the end of the interview. On the other hand, many PLS expressed an understanding for the way other people behaved towards them. Several appeared to endorse the negative reactions, or, at least, did not see them as unfair. For example, PLS stated that others were *‘right’* in making critical comments, and that it was their own responsibility to try harder to get better and control *‘incorrect’* behaviours.

In response to the question ‘how should others behave towards you?, a woman from Satara answered:

*“Just as they behave now. ( … ) I should recover soon and they should not tell me anything, I should understand it myself. ( … ) I feel I should be good, I should improve. I should become just like other people are. I should behave well.“*

#### 3.2.2 Anticipated discrimination/*Anticipated reactions*

Just over half (52%) of the participants reported that they had stopped themselves from taking up opportunities in the past 12 months, because they anticipated negative reactions from others. The proportion reporting anticipated discrimination on each item is given in Fig. S2 (Online file C).

In qualitative interviews, anticipated negative reactions emerged as a source of great concern to many interviewees. As a consequence, they would often avoid contact with others. Among their concerns were worries that other people would *‘spread the news’*, *‘look down upon them’*, *‘tease them’*, *‘treat them without respect’*, *‘blame them’* or *‘avoid them’*. PLS were also concerned about *‘negative impact on marriage or work’*, both for themselves and for other family members. Sometimes, PLS were so concerned about possible negative reactions that they avoided using treatment facilities for fear that others would find out about their condition. A young man from Goa explained:

*“If somebody sees me [when I go to see the psychiatrist, they will pass the news that I am not well. My name will be spoilt. My life will be full of changes. Nobody will come close to me, everybody will avoid me. Because this illness is like that. Everybody avoids people having this type of illness.”*

#### 3.2.3 Discomfort to disclose the illness/*Others finding out*

Almost half (46%) of the quantitative sample said they were ‘uncomfortable’ or ‘very uncomfortable’ about disclosing their illness. In the in-depth-interviews, most interviewees stated they preferred that other people did not know about it because of the anticipated negative reactions or because disclosure would make them feel inferior or ashamed. Many made efforts to evade *‘others finding out’* by avoiding contact or giving alternative explanations when they were seen on the way to a treatment facility. Commonly, others found out about the condition through observed behaviour or hearing from others (*‘gossip’*) rather than active disclosure. It was striking that family caregivers were often keen to keep the illness a secret and would sometimes instruct the PLS not to speak to others or stay inside the house. Disclosure was of particular concern for unmarried PLS and was usually kept a secret from future spouses and in-laws. A woman from Satara explained:

*“[If my husband had known] he would not have gotten married to me. If he had been told, he would have said “if she is with me, how will she look after me, will she prepare food for me? When she becomes a mother, will she look after the children?”*

#### 3.2.4 Sense of alienation/*Negative views and feelings about the self*

The proportion of participants reporting a sense of alienation was relatively high: 79% reported any alienation, i.e., they agreed or agreed strongly with at least one item of the alienation scale. Close to 40% had an Alienation Mean Score of >2.5, i.e., they agreed, on average, with all the statements of the alienation scale. (‘High alienation’ (Ritsher et al., 2003)). The proportion agreeing with a given item is illustrated in Fig. S3 (Online file D).

Qualitative findings reported here refer to the wider domain of negative views and feelings about the self, which includes feelings of alienation. In in-depth-interviews, low self-esteem and feelings of inferiority due to the illness were salient themes. Interviewees would usually compare themselves unfavourably with others in regard to achievements in important areas of life, e.g., unemployment, not being married, or not having money. Some also perceived their own behaviour as inadequate or felt inferior because others knew about their illness.

PLS were often self-critical and would use negative terms to describe themselves, e.g. saying *‘I am weak’*. A few would blame themselves for having brought about their illness, criticise themselves for being *‘stubborn’* or *‘lazy’*, or speak regretfully about their *‘incorrect’* behaviours, implying that it was their responsibility to change. Many seemed to be painfully aware of the burden they were imposing on their family members.

A few spoke explicitly about ‘*feeling different’* from others or said they had been *‘separated’*. A woman from Tamil Nadu explained:

*“Since I am like this [my family members] are behaving like this ( … ). To me only it happened, God separated me. ( … ) God did not keep me like all others, that is why they are talking like this. ( … ) God rejected me ( … ) [my sisters] are all married and well settled, but God separated me, you know; now I am like this.”*

### 3.3 Factors influencing experiences of stigma and discrimination

The second objective of the study was to identify factors influencing PLS’ experiences of stigma and discrimination. We describe quantitative findings on the hypothesised association between symptom severity and negative discrimination and results of multivariate regression modelling of negative discrimination. In qualitative data, factors shaping experiences of stigma were explored by investigating the links connecting the key theme of ‘negative reactions and changes in relationships’ with other themes that emerged from thematic data analysis (Fig. 2).

To investigate determinants of negative discrimination quantitatively, we examined the hypothesis that PLS with higher levels of symptom severity had higher levels of negative discrimination. Contrary to our prediction, PANSS total symptom score was not associated with negative discrimination. However, the PANSS subscales on positive and negative symptoms were significantly associated with levels of negative discrimination, but each influenced the outcome in opposite directions: for each step up to the next higher quartile of the PANSS positive symptom score, the odds of experiencing negative discrimination *increased* on average by 24% (Crude OR = 1.24; 95% CI 1.00–1.54; *p* = 0.05). For each step up to the next higher quartile of the PANSS negative symptom score, the odds of experiencing negative discrimination *decreased* on average by 24% (Crude OR = 0.76; 95% CI 0.61–0.94; *p* = 0.01).

Further analyses were undertaken to identify the specific symptoms most clearly associated with the outcome. Levels of negative discrimination were significantly associated with ‘delusions’ (*p* < 0.01) and ‘suspiciousness’ (*p* < 0.01) and, inversely, with ‘poor rapport’ (*p* = 0.01) and ‘lack of spontaneity or flow of conversation’ (*p* = 0.02).

A multivariate regression model confirmed the roles of positive and negative symptoms as independent determinants of negative discrimination even after adjusting for other determinants, and further revealed that negative discrimination was independently associated with higher caregiver knowledge about symptomatology, lower participant age and not having a source of drinking water in the home (Table 2; Online file B).

To explore factors shaping experiences of ‘negative reactions’ in qualitative data, we explored the links connecting the themes that emerged from the analysis:

• *‘Behaviours and manifestations of the illness ‘* (Theme I)
• *‘Reduced ability to meet role expectations and personal aims’* (Theme II)
• *‘Others finding out’* (Theme III), including the subtheme *‘anticipated reactions’*
• *‘Negative reactions towards the PLS and changes in relationships’* (Theme IV), including the subtheme *‘negative reactions’*
• *‘Negative views and feelings about the self’* (Theme V)

The themes were linked to each other in multiple ways, which are summarised graphically in Fig. 2. We only discuss here the links between themes I, II, III and V and ‘negative reactions and changes in the relationships’ (Theme IV) – the domain corresponding most closely to the concept of ‘negative discrimination’ –to provide context for the quantitative findings.

#### 3.3.1 Key link 1 (Arrow 1, Fig. 2)

‘*Negative reactions from others and changes in relationships’* (Theme IV) were commonly linked to *‘Others finding out’*. One of the main reasons for interviewees' concerns about others finding out were the negative reactions they had experienced or anticipated. Certain forms of negative reactions, e.g., *‘teasing’* or *‘being called mad’,* were directly linked to others having ‘found out’. Furthermore, most participants' voiced fears that disclosure would affect marital prospects of PLS or other family members or work prospect for the PLS (Arrow 6), and thereby lead to social devaluation (Arrow 3). In other words, ‘*Others finding out’* was such an important concern largely *because* of the impact it was seen to have on ‘*PLS’ ‘ability to meet role expectations’*, and thereby also other family members' ability to meet their own role expectations, e.g., a parents ability to *‘discharge their duty’* of marrying their children off, which would lead to public disapproval and shame for caregivers and PLS alike. Tensions around the domain of *‘Others finding out’* therefore affected interactions within the family; indeed, some of the restrictions PLS experienced, e.g., being stopped from going out, were attempts by caregivers to conceal the illness.

#### 3.3.2 Key link 2 (Arrow 2)

Both PLS’ and caregivers' narratives further described a clear link between the PLS's *‘Behaviour and illness manifestations’* and the negative reactions PLS faced from others (Arrow 2). The types of behaviour most frequently named in this context were disruptive or aggressive behaviour, ‘laziness’ or ‘not working’, odd behaviour in public, inappropriate talk, poor self-care or inappropriate dress. The link was particularly evident in regard to angry reactions, e.g., critical comments and physical aggression, and to experiences of restrictions or emotional distancing by family members.

PLS often saw other people's reactions towards them as conditional upon their behaviour. A woman from Satara explained:

*I: Does [your sister-in-law] talk with you?*

*P: Yes. She does [now]. I do not behave as madly as in the past. I watch television, cook food, etc. So she does not say anything [critical] to me.*

#### 3.3.3 Key link 3 (Arrow 3)

Finally, experiences of negative reactions and social devaluation were often associated with participants *‘reduced ability to meet role expectations’*.

Within families, this was particularly evident in PLS being scolded, shouted at or even beaten because they were not going for paid work or failed to keep up with household duties. Some PLS also experienced critical comments, including from relatives and neighbours, for being financial burdens or making it difficult for siblings to marry.

*My mummy and my daddy they all shout at him [the PLS]. Because he is not doing anything, no, not doing work and all. It is natural, no, if you are not doing work ( … ) anybody will shout ( … ). Just eating and sleeping means there is no meaning only, no?*

*Female caregiver, sister of male PLS, Goa*

PLS themselves would often attribute the negative reactions they experienced to their reduced ability to meet role expectations in key areas of life. For example, a young man from Goa, explained that all his friends had left him not because of his ‘illness’, but because he was now unemployed and did not have money. A man from Satara explained that it was only understandable that he was criticised at work, seemingly not expecting allowances to be made for his illness. Correspondingly, PLS did not usually portray negative responses towards them as ‘unfair’, but would instead show understanding for other people's reactions, and try even harder to comply with expectations.

## 4 Discussion

To the best of our knowledge, this research represents one of the largest mixed-methods studies of subjective experiences of stigma and discrimination faced by PLS in a LAMIC setting.

### 4.1 Integrated descriptive findings

Quantitative findings on four different measures showed that internalised forms of stigma experience were reported more commonly than actual negative discrimination.

Alienation (reported by 79%) and anticipated discrimination (reported by 52%) were particularly common, and related themes were salient in qualitative interviews. Low self-esteem, self-criticism, self-blame and feelings of alienation were clearly evident in in-depth-interviews and emerged as important factors influencing PLS’ overall emotional wellbeing. There was relatively good consistency with findings of other studies measuring internalised or anticipated stigma using the ISMI, the anticipated discrimination subscale of the DISC or other measures (Ritsher et al., 2003, Thornicroft et al., 2009).

Negative discrimination, on the other hand, was reported less commonly (42%) than aspects of internalised stigma, and much less commonly than in other studies that have employed the DISC. This might in part be explained by the reference time frame of 12 months used, i.e., participants might have experienced more discrimination in earlier stages of their illness. Yet, even when comparing to studies using the same 12-month time frame, our findings indicated very low levels of negative discrimination (42% compared to 91% in a recent United Kingdom sample) (Henderson et al., 2012). Qualitative data, however, do not support an interpretation of this finding as indicating that PLS did not experience negative reactions that would have led to feelings of devaluation and distress. Rather, they suggest that the specific aspect of discriminatory experiences elicited by the DISC, i.e., treatment perceived as unfair and attributed to the illness, constituted only one of several forms of negative reactions that participants experienced. For example, qualitative interviews highlight that PLS experienced negative reactions, e.g., avoidance or critical comments, but did not necessarily perceive these as 'unfair' – rather, some interviewees expressed understanding of other people's reactions towards their disability and would take upon themselves the responsibility of changing whatever they saw as the mark that set them apart from others. In other cases, interviewees attributed negative reactions not to having an illness (Key link 1), but rather to what they saw as 'incorrect' behaviour (Key link 2) or reduced ability to meet role expectations, such as being unemployed or unmarried (Key link 3).

#### 4.1.1 Less stigma in India?

While the observation of low negative discrimination rates in this study needs to be acknowledged, it is important to keep in mind that ‘experiences of negative discrimination’ (as defined and measured by the DISC) represent just one domain of the complex construct of ‘stigma’ and its effects on individuals. The findings from the other stigma measures used in this study, qualitative interviews, and existing research from India present a somewhat different picture, and therefore, do not justify the conclusion that the impact of stigma associated with schizophrenia is less severe or problematic in India.

Overall, stigma and discrimination tended to manifest with an ‘internalised’ phenotype for the participants of this study, i.e., concerns about ‘others finding out’, worries about what *might* happen in interaction with others, perceptions of being ‘looked down upon’ or feelings of low self-esteem, shame and alienation emerged as more dominant manifestations of stigma than the negative reactions actually enacted by others. Fitting the notion that internalised stigma (or ‘self-stigma’) represents the opposite of empowerment (Corrigan and Rao, 2012), PLS in this sample often appeared disempowered, with low expectations for themselves and a sense of ‘perceived legitimacy’ (Rusch et al., 2006) of being treated as someone of lesser status. Internalisation of stigma may further have contributed to the relatively low levels of negative discrimination reported, as participants avoided contact with others due to fears of disclosure, shame and anticipated discrimination, or were less likely to view the reactions of others as discriminatory or unfair. Many participants had small social networks, and negative reactions, if any, were mostly from sources close to the PLS, on quantitative and qualitative measures, alike.

### 4.2 Integrated findings on factors influencing experiences of stigma

We discuss here three key factors that emerged as particularly important in determining stigma experiences in this study.

#### 4.2.1 Symptoms of schizophrenia

Both in quantitative and qualitative data, a clear link between symptoms/illness manifestations and the experience of negative discrimination/negative reactions was evident.

Findings of hypothesis testing indicated that symptom severity did predict PLS’ experience of negative discrimination, however, not in the way originally postulated. Rather than overall symptom severity (PANSS total symptoms score), certain illness features, namely higher levels of positive symptoms (PANSS positive symptoms score) and lower levels of negative symptoms (PANSS negative symptoms score) were significantly associated with the odds of experiencing negative discrimination. The role of positive and negative symptoms became even more clearly evident once other predictors were entered into the multivariate model (Table 2; Online file B). In qualitative data, ‘positive symptoms’, e.g., ‘*disruptive*’ or *‘odd behaviour’* and specific ‘negative symptoms’, e.g., reduced self-care, were positively associated with negative reactions (see Fig. 2, Arrow 2). *‘Behaviours and illness manifestations’* were also important because they often determined *‘Other people finding out’* and’ ‘*Reduced ability to meet role expectations’* (see Fig. 2, Arrows 4 and 5.)

The finding, in univariate analyses, that two of the symptoms most clearly associated with negative discrimination were ‘delusions’ and ‘suspiciousness’, suggests that the association between symptoms and discrimination may have been confounded by the nature, rather than the severity of symptoms. However, we consider it unlikely that paranoid misinterpretation would explain a large part of the discrimination reported given: i) the qualitative finding that illness manifestations were linked to negative reactions in both PLS' and their caregivers' accounts of negative reactions towards the PLS, ii) studies to show that diagnosis was not associated with rates of negative discrimination (Farrelly et al., 2014), iii) evidence supporting the association from other settings (Livingston and Boyd, 2010) and in studies from India (Charles et al., 2007, Loganathan and Murthy, 2008). One Indian study specifically identified suspiciousness and paranoid behaviour as a cause for avoidance by neighbours and embarrassment among caregivers of PLS (Raguram et al., 2004).

#### 4.2.2 Others finding out

In keeping with the labelling theory of stigma (Link and Phelan, 2001), qualitative findings further identified a link between *‘Others finding out’* (whether and how much other people knew about the condition and labelled the PLS as being ‘ill’ or ‘different’) and negative reactions (Arrow 1; Fig. 2). *‘Others finding out’* was often a consequence of people observing visible illness manifestations (Arrow 4), and derived much of its importance due to concerns about impact on ability to meet role expectations (Arrow 6).

#### 4.2.3 Reduced ability to meet role expectations in marriage and work

The qualitative study findings also highlight a link between PLS’ ‘*Reduced ability to meet role expectations’* in terms of work, marriage or financial standing, and the negative reactions experienced (Arrow 3; Fig. 2). There may be both socio-economic and cultural factors contributing to this phenomenon:

Hindu philosophy, which has been influential on Indian society across religious groups, holds that doing one's duty in life (living in accordance with ‘*Dharma*’) is central to a moral life (Avasthi et al., 2013) and that living by the ways of conduct described by *Dharma* (i.e., meeting social role expectations and codes of behaviour) will lead to purification of mind and, ultimately, *Moksha* (liberation) (Srivastava et al., 2013). Furthermore, it is necessary to consider that aims such as employment hold added importance in LAMIC settings with minimal state welfare provisions, where loss of income from PLS may constitute an existential threat to the economic survival of the entire family. This is particularly relevant in the case of schizophrenia which affects young adults in the most productive periods of their lives. In India, there are, typically, clearly demarcated roles for men and women, with women being expected to be chiefly concerned with family and household duties whereas men bear the financial responsibility for the household and hold the main decision-making power (Avasthi et al., 2013, Loganathan and Murthy, 2008). Consequently, unemployment and underachievement pose huge threats to a man's social status (Thara et al., 2004) and not getting married constitutes an existential risk for women (Thara and Srinivasan, 1997). A number of studies discuss the specific importance of marriage in Indian society: as a desired outcome, an economic necessity, a social role expectation, and a potential ‘cure’ for mental illness (Hopper et al., 2007, Thara and Srinivasan, 1997, Weiss et al., 2001). According to one study, women with schizophrenia and broken marriages perceived the loss of social status associated with a broken marriage as *more* burdensome than the stigma associated with their mental illness (Thara et al., 2003). Supporting the above, formative research for COPSI found that ‘employment’ and ‘fulfillment of duties and responsibilities’ were named among the highest priority outcomes for PLS and caregivers (Balaji et al., 2012b, Balaji et al., 2012a).

#### 4.2.4 What ‘matters most’?

In summary, the findings of this study suggest that what ‘matters most’ to the moral status of PLS in India is to be able to meet gender-specific role expectations with regard to work and marriage, and adhere to codes of conduct in terms of socially acceptable behaviour. The salience of work and marriage is enhanced through the existential economic importance of achieving these aims in the context of poverty and the virtual absence of social welfare benefits. Due to the high levels of family cohesion, the damaging effects of stigma in India tend to exert their effects on entire families. This means that – much as has been described for the context of China - what is ‘at stake’ through the stigma of schizophrenia in India is not only the wellbeing and status of an individual PLS but the status of a family lineage for generations to come (Yang et al., 2014a, Yang et al., 2014b, Yang et al., 2007).

### 4.3 Study limitations

Despite efforts to make the study sample representative, it only includes PLS in psychiatric care, and it is possible that untreated cases may have reported different experiences of stigma and discrimination. Potential limitations also relate to the diagnostic eligibility for the study, which was determined by treating psychiatrist rather than researchers, and the fact that the qualitative sample was purposively selected to over-represent PLS with higher levels of negative discrimination.

We have further elaborated on the need to consider context-specific factors when measuring subjective reports of ‘stigma and discrimination’, particularly with regard to measurements requiring normative judgements, such as ‘fair’ or ‘unfair’, and have sought to address this through triangulation in the mixed-methods approach adopted. Language and cultural barriers, and the fact that many study collaborators were psychiatrists, may also have played a role in data analysis and interpretation. Finally, it is possible that the desire not to speak negatively about family members or the care received may have led to social desirability bias.

### 4.4 Implications

The findings of this study have implications for research and for interventions to reduce the impact of stigma. They illustrate that ‘experiences of stigma and discrimination’ are shaped by context-dependent factors, and that stigma research and interventions need to address this in their design (Yang et al., 2014a, Yang et al., 2014b). For the Indian context, our findings suggest the need to recognise that: i) the meaning of stigma is determined by what ‘matters most’, i.e. in India, the threat to PLS’ ability to meet role expectations and codes of behaviour, and the impact on the social status of entire families ii) negative social reactions to mental illness may not be viewed as ‘unfair’ iii) internalised, anticipated and perceived stigma may result in considerable distress, even in the absence of actual experiences of discrimination iv) family members and neighbours provide most social contact, and negative reactions enacted by family members and sources close to PLS need to be addressed in interventions.

From a conceptual point of view, qualitative findings further raise the hypothesis that not one, but several illness-related attributes, e.g., known-about illness (Arrow 1, Fig. 2), socially unacceptable behaviour (Arrow 2) and reduced ability to meet role expectations (Arrow 3), with context-dependent meanings are relevant in the generation of negative social reactions towards PLS. Further research is required to study the nature and cumulative effects of these overlapping pathways in order to identify access points for interventions.

Notably, to the PLS who participated in this study, it did not seem to matter whether negative societal reactions arose through associations with an illness label (Arrow 1, Fig. 2) (the pathway most clearly linked to classical notions of 'stigma and discrimination' (Link and Phelan, 2001)) or through another pathway. From a healthcare perspective, more important than asking ‘how can we reduce stigma and discrimination associated with mental illness?’ may therefore be the question ‘how can we reduce the frequency, intensity and impact of negative social responses towards PLS (which may be partly, but not necessarily entirely mediated through ‘stigma’)?

A number of implications arise for practice and stigma interventions.

Psychosocial healthcare interventions for schizophrenia should adopt as one of their aims to reduce the effects of societal stigma on PLS, informed by the perspective of 'what matters most' to people's sense of worth and social acceptance in their local context. Based on the findings of this study, this should include, for India: i) treatments that help PLS manage the types of illness manifestations most clearly associated with negative reactions (e.g., ‘positive symptoms’, reduced self-care), ii) recovery-oriented work that supports PLS in taking up social roles that fulfil them and earn them respect from others (particularly in regard to work and marriage) and iii) interventions that help empower PLS and overcome feelings of alienation and low self-esteem. The latter could comprise the involvement of PLS in stigma interventions, support groups where PLS can share experiences and coping strategies, or peer-support interventions (Corrigan and Rao, 2012). Furthermore, research should evaluate how psycho-social care interventions can be enhanced in context-appropriate ways to offer: iv) support to PLS dealing with negative social reactions, including from family members; and v) support with disclosure decisions (Corrigan and Rao, 2012). Acknowledging the important role of family members as caregivers, people affected by stigma and people enacting stigma, interventions need to adopt a systemic approach that involves family members as appropriate.

The high levels of perceived and anticipated stigma reported also illustrate that efforts to reduce stigma at the community level are required. Our findings suggest that such interventions should emphasize recovery-oriented messages, the message that PLS lead meaningful lives, and promote tolerance for alternative models of life outside the typical social script of traditional roles in work and marriage. Finally, findings highlight the need for structural changes that provide support to PLS facing disability or discrimination, e.g., in the form of unemployment and incapacity benefits, benefits for divorced women, disability legislation and services and importantly, effective and accessible healthcare (Thornicroft, 2006, Yang et al., 2014a, Yang et al., 2014b).
