# Supplementary material for: Experiences of stigma and discrimination of people with schizophrenia in India
Source: Soc Sci Med. 2014 Dec;123:149–59. doi: 10.1016/j.socscimed.2014.10.035 (PMC4259492; doi:10.1016/j.socscimed.2014.10.035)
Supplement: Supplementary file 1 [file mmc1.pdf]

# Interview guide for semi-structured in-depth-interviews with people living with schizophrenia

Community Care for People with Schizophrenia in India (COPSI), 2009

## Introduction

I would like to start by asking you a little about yourself

- Can you tell me a little about yourself?
- What is your day like?
[*Probe:* occupation; routine; interests; who is at home]

## Illness Onset and Explanatory Models

- Can you tell me about the problems you are seeing Doctor X [treating psychiatrist] for/ you are taking medicines for?

  - When did you first notice these problems?
  - How did the problems start/ What happened? [establish onset of illness/problems]
  - How did your family members react? *(explore individual reactions to the manifestation of illness or diagnosis)*
  - What did you do? What did your family do? [establish process of helpseeking, approximate course of illness]

- What do you think has happened to you?

- What do you call these problems?
*[Probe: if mentions a term like 'schizophrenia'*: what do you mean when you say this?]

- What do you think has caused these problems?
[*Probe if necessary:* something that happened to you life events, stress, upbringing; something to do with yourself; anything else]

- What do others say is the reason this has happened?
[*Probe individually for:* Family members, friends
Be attentive to any evidence of others blaming the patient for the illness and ask about it]

## Impact on Home/Family life

- **Can you tell me about your life at home?**

  *[Probe for:]*
  - Routine [e.g. please tell me about a typical day at home]

- Involvement in family activities
    - Economic contribution
    - Household work

*[If speaks about doing less work, explore]:*

- how do your other family members react to this?

- Relationships with other family members (parents, siblings, spouse, children)

- Meal times [Eating at the same time as others? Sitting with others?]

- Involvement in decision making in family

- Taking on/being given responsibilities (incl. taking care of children, if applicable)

- Visitors coming to the house [Being introduced to others? Sitting with others?]

- Going to Family functions/ Weddings

- Taking part in religious rituals in the home

- Going to Temple/ Church with family members

    *[Explore: Interaction with others while going out: Do they speak to him/her? How do they behave towards him/ her? How do family members react in that situation?]*

- Going out (going to shops, taking the bus, etc.)

    - *[Explore: Interaction with others while going out, do they speak to him/her? How do they behave towards him/ her? How do family members react in that situation?]*

- Recreational activities at home (e.g. watching TV)

*[For each Probe explore:]*

- How is it now?
- How was it before the illness/problems started?
- What has changed? Can you give me examples?
- Why you think this is happening/ it has changed?
- How do you feel about it?

- Can you tell me about good times you have (in the family, with friends)?

  *[If speaks about a 'good time' in the past:]*
    - What about now? Are you happy?

  *[Or ask generally:]*
    - Do you feel happy?

- Can you tell me about a difficult situation that has occurred at home? *[probe for example:* disagreements about activity levels, household work, education/work, marriage]

**Other areas of impact**

*[Probe area of impact]:*

- Relationships with relatives: closeness, frequency of seeing them
- Friendships: closeness, supportiveness
- Intimate relationships: interested/not interested; sustained or not
- Education, work: unable to perform, concentrate, continue work (including employment)
- Finances: able/unable to afford treatment

*[Guidelines for each probe]*

- How is it now?
- How was it before the illness started?
- What has changed? Can you give me examples?
- Why do you think it has changed?
- How do you feel about this?

- What else has changed since the illness started?/ How (else) has the illness affected your life?
  - Can you give me examples?
- What about some good changes? Have there been any?
  - Can you give me examples?

**Other people knowing and treatment from others:**

- **Do others know about your illness/problems?**

  ***[If no]***

  - Why do they not know?

  *[If prefers to hide illness]*

  - Who should not know?

    *Probe:*

    - *Other family members (relatives),*
    - *friends,*
    - *neighbours,*
    - *employers/people at educational institution*

  - Why do you feel this way?
    *[Probe:* fear of rejection, ridicule, being treated differently]
  - What do you think would happen if they knew?
  - Has anything like that happened to you?
  - What do you do to make sure other people don't find out about the illness/problems?
  - When people ask you what has happened to you, what do you tell them?

  ***[If yes]***

  - Who knows?
  - How did they find out?
  - How did they react?

  *[Ask even if everybody knows about illness]:*

  - How do you feel about them knowing about it?
  - Would you prefer some people not to know?/Who should not know? Why?

- How do they treat you? Can you give me examples?

  *Negative: treating differently, lack of respect, ridiculing, rejecting*
  *Positive: supportive, understanding, considerate*

  *[Probe, also if these people may not know about the illness, but clarify this if necessary]*

  - Friends,
  - Wider family (relatives),
  - Neighbours
  - People at educational institution; at work
  - Paid help in the house
  - People at the Temple/ Church?
  - People in the street or on the bus?

  How do others treat you when it comes to
  - Going to social functions (weddings, etc)?
  - Marriage proposals?

> *[Guidelines for each probe]*
>
> - How do they treat you?
> - How did they treat you before the illness started?
> - What is different now/ What has changed?
> - Why do you think this is?
> - How do you feel about this?

- How should people treat you? How would you like people to treat you?
  - Why do you feel this way?

**Coping**

*[Refer to negative experiences that have been reported:]*

- What do you do when people treat you in this way?
- How do you react?
- How do you manage? What helps you when this happens?
- Who helps you?
- With whom do you share your feelings?

**Experiences with treatment so far:**

I would now like to ask you a little about your treatment

- What has your experience been with the treatment you have been getting so far? *[probing for treatments received so far that were mentioned earlier]*

    - What did you like about it? What did you not like?
    - How do you feel about the treatment you are getting at the moment?
    - How is it for you when you go and see your doctor?

    *[Probe for: interaction with doctor; feeling respected; worries about being* seen when going]

- What do your family members say about the treatment you are taking? How do they react?

**Beliefs about the Illness**

- Do you know anyone else who is having similar problems to yours?
    - Can you tell me about it?

*[Probe: what do you think happened to him/her?/Still in contact or not/Attitude towards this person]*

- What do you think other people say/think about people who have this illness?
    - How do they react?
    - How do they treat them?

    *[If negative beliefs are expressed:]*
    - What is it exactly that is not so good about this illness?

- Suppose you had diabetes rather than this illness, how would that be? *(explore whether and in what way that would be different)*

    *[If engaging well with the above:]*

    - What if you had epilepsy rather than this illness, how would that be?
    *(explore whether and in what way that would be different from their current problem)*

    *[If the above phrasing seems too direct to ask the participant, try the following: ]*
    - Suppose there is one person who has a similar problem to yours and another person who has diabetes:
    - Will people treat him/her the same or different?

*If different:*
  - Why?
  - What is it about the illness that is different?

- Have you heard of the term 'discrimination'?
  - What does it mean to you?
  - Can you give me an example?

  *If another example than mental illness as a reason for 'discrimination' is given, explore:*
  - Some people tell me that people with health problems like yours are sometimes treated differently – what do you think?
  - Has anything like that happened to you?

**Impact on self-esteem and confidence**

- How do you feel about yourself?
  *[Probe:* feeling good/bad about self; confident/less confident; shame, embarrassment:]

  - How was this before the illness started?
  - What is different now/ What has changed?
  - Why do you think this is?

**Future:**

Lastly, I would like to ask you a little about the future

- What are your hopes for the future? Can you tell me about it?

  - How will you achieve this?
    *[Probe:* confident/ not so confident will achieve this, why]

- What changes would you like to make in your life?

*Probe for:*
  - improved relationships,
  - Resuming routines, work, education,
  - marriage
  - building up social life/friendships
  - independence
  - no or less symptoms
  - any other themes that came up earlier

*[Guidelines for each probe]*

- What sort of changes do you want in this?
- Why do you want this?

*[Explore for themes related to marriage, work, education and where else appropriate:}*

- How will your life be different if this happens?

**Closing questions:**

We have reached the end of the interview. Is there anything more you'd like to tell me that we have not already discussed?

Do you have any questions for me about anything we discussed?

**Thank you again for your time and for allowing me to talk to you. You have been very helpful to us.**

**-- End of interview--**
