# Supplementary material for: Experiences of stigma and discrimination of people with schizophrenia in India
Source: Soc Sci Med. 2014 Dec;123:149–59. doi: 10.1016/j.socscimed.2014.10.035 (PMC4259492; doi:10.1016/j.socscimed.2014.10.035)
Supplement: Supplementary file 2 [file mmc2.pdf]

Online table 1: Crude and adjusted odds ratios of factors independently associated with negative discrimination

Exposure variable	Crude OR	95% CI	P	Adj. OR[1]	95% CI	P
**PANSS positive symptom score subgroups - linear effect** (average OR for each step up in positive symptoms score (quartiles), assuming linearity[2])	1.24	1.00 – 1.54	**0.05**	1.36	1.07 – 1.73	**0.01**
**PANSS negative symptoms score subgroups - linear effect** (average OR for each step up in negative symptom score (quartiles), assuming linearity[2])	0.76	0.61 – 0.94	**0.01**	0.68	0.54 – 0.87	**<0.01**
**Caregiver knowledge about symptomatology**						
Relatively low (score 1 or 2)	1			1		
Relatively high (score 3 or 4)	1.88	1.15 – 3.06	**0.01**	2.14	1.26 – 3.65	<**0.01**
**PLS age** (average OR for each step up in age group (10 years), assuming linearity[2])	0.75	0.59 – 0.94	**0.01**	0.70	0.55 – 0.89	**<0.01**
**Source of drinking water**						
Tap water (in the house)	1			1		
Other water source (public tap; well water; any other source)	1.55	0.95 – 2.51	**0.08**	1.84	1.07 – 3.14	**0.03**

[1]Adjusted for all other exposure variables included in the final model (listed in the first column of the table)
[2]Likelihood ratio tests indicate there is no good evidence against the assumption of linear trend p>0.05

Factors independently associated with negative discrimination (online table 1):

The multivariate regression model presented in online table 1 indicates that the experience of any negative discrimination in the last 12 months was independently associated with higher PANSS positive symptom score, higher caregiver knowledge about symptomatology, lower PANSS negative symptom score, lower participant age and not having a source of drinking water in the home.

The association of negative discrimination with positive and negative symptoms of schizophrenia is discussed in detail in the 'Results' and' Discussion' sections of the main paper.

Other associations identified in online table 1 are more difficult to explain. Lower participant age might increase chances of negative discrimination because, according to qualitative findings, younger PLS tended to have higher levels of social interactions and spend more time in the public. The association of source of drinking water with negative discrimination may represent a chance

finding, however, a possible interpretation could be that not having a source of drinking water in the home was a proxy measure for ways of life involving high levels of community interaction, e.g., fetching water might have rendered participants more exposed to negative reactions from others. Finally, the association between higher caregiver knowledge of schizophrenia and negative discrimination gives rise to the question whether lower levels of 'knowledge about schizophrenia' reflected in fact a dominance of non-medical explanations of the 'problem'. This hypothesis is supported by a growing body of research indicating that certain forms of knowledge, particularly information projecting a biomedical model of mental illness, may increase rather than decrease social distance from people with mental illness(Phelan, Yang, & Cruz-Rojas, 2006; Schomerus et al., 2012; Yang et al., 2011).

Phelan, J. C., Yang, L. H., & Cruz-Rojas, R. (2006). Effects of Attributing Serious Mental Illnesses to Genetic Causes on Orientations to Treatment. *Psychiatric Services, 57*(3), 382-387.

Schomerus, G., Schwahn, C., Holzinger, A., Corrigan, P. W., Grabe, H. J., Carta, M. G., et al. (2012). Evolution of public attitudes about mental illness: a systematic review and meta-analysis. *Acta Psychiatrica Scandinavica, 125*(6), 440-452.

Yang, L. H., Lo, G., Wonpat-Borja, A. J., Singla, D. R., Link, B. G., & Phillips, M. R. (2011). Effects of labeling and interpersonal contact upon attitudes towards schizophrenia: implications for reducing mental illness stigma in urban China. *Social Psychiatry & Psychiatric Epidemiology*.
