# Supplementary figures and images for: Experiences of stigma and discrimination of people with schizophrenia in India
Source: Soc Sci Med. 2014 Dec;123:149–59. doi: 10.1016/j.socscimed.2014.10.035 (PMC4259492; doi:10.1016/j.socscimed.2014.10.035)

**Online figure 1: Anticipated discrimination item percentages**

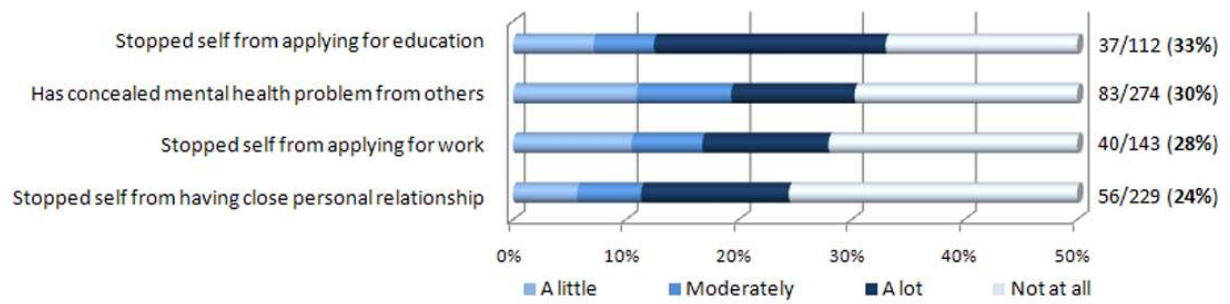

Supplement: Supplementary file 3 [file mmc3.pdf]

**Online figure 2: Alienation item percentages**

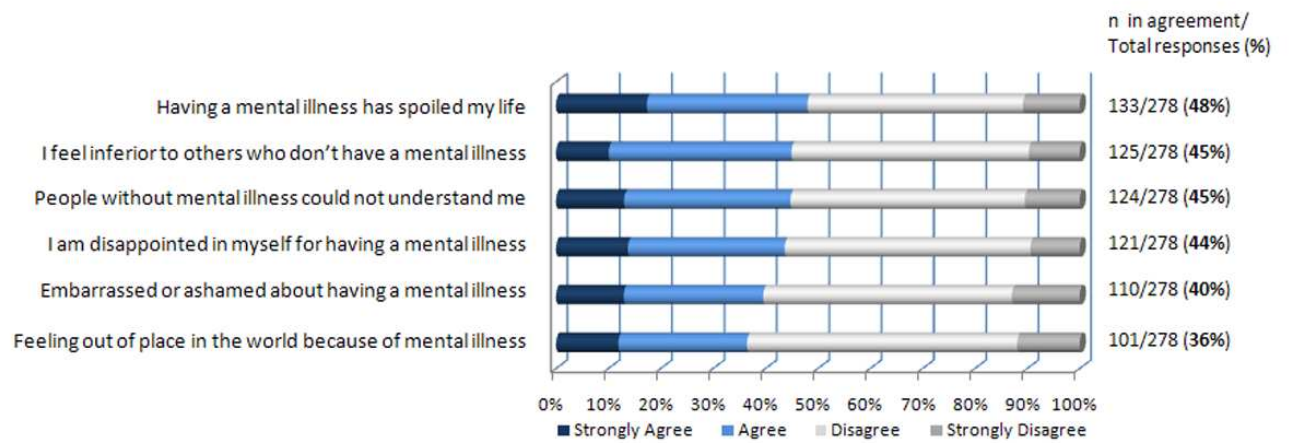

Supplement: Supplementary file 4 [file mmc4.pdf]
